# Femoral Tunnel Position Affects Postoperative Femoral Tunnel Widening after Anterior Cruciate Ligament Reconstruction with Tibialis Anterior Allograft

**DOI:** 10.3390/jcm12051966

**Published:** 2023-03-02

**Authors:** Sung-Sahn Lee, Il Su Kim, Tae Soo Shin, Jeounghun Lee, Dae-Hee Lee

**Affiliations:** 1 Department of Orthopaedic Surgery, Ilsan Paik Hospital, Inje University School of Medicine, Goyangsi 10380, Republic of Korea; 2 Department of Orthopaedic Surgery, Samsung Medical Center, Sungkyunkwan University School of Medicine, Seoul 06351, Republic of Korea

**Keywords:** anterior cruciate ligament, reconstruction, tunnel widening, risk factor, laxity

## Abstract

This study aims to identify potential factors for both femoral and tibial tunnel widening (TW) and to investigate the effect of TW on postoperative outcomes after anterior cruciate ligament (ACL) reconstruction with a tibialis anterior allograft. A total 75 patients (75 knees) who underwent ACL reconstruction with tibialis anterior allografts were investigated between February 2015 and October 2017. TW was calculated as the difference in tunnel widths between the immediate and 2-year postoperative measurements. The risk factors for TW, including demographic data, concomitant meniscal injury, hip–knee–ankle angle, tibial slope, femoral and tibial tunnel position (quadrant method), and length of both tunnels, were investigated. The patients were divided twice into two groups depending on whether the femoral or tibial TW was over or less than 3 mm. Pre- and 2-year follow-up outcomes, including the Lysholm score, International Knee Documentation Committee (IKDC) subjective score, and side-to-side difference (STSD) of anterior translation on stress radiographs, were compared between TW ≥ 3 mm and TW < 3 mm. The femoral tunnel position depth (shallow femoral tunnel position) was significantly correlated with femoral TW (adjusted *R*^2^ = 0.134). The femoral TW ≥ 3 mm group showed greater STSD of anterior translation than the femoral TW < 3 mm group. The shallow position of the femoral tunnel was correlated with the femoral TW after ACL reconstruction using a tibialis anterior allograft. A femoral TW ≥ 3 mm showed inferior postoperative knee anterior stability.

## 1. Introduction

Tunnel widening (TW) after anterior cruciate ligament (ACL) reconstruction is a well-known phenomenon. The incidence of TW after single-bundle ACL reconstruction is reportedly between 30.1–100% on the femoral side and 20.9–73.9% on the tibial side [1,2,3]. TW might lead to two-stage surgery in revision ACL reconstruction [4,5]. Moreover, TW adversely affects patients’ postoperative outcomes [6,7,8].

Several studies have attempted to identify the risk factors for TW [3,9,10,11]. It is generally accepted that TW is caused by a complex interplay between biological and mechanical factors. The biological factors include patient age, bone quality, cell necrosis induced by drilling, or inflammatory mediators [12,13,14,15,16]. Mechanical factors include the femoral fixation method, tibial slope, graft position, graft tension, and aggressive rehabilitation [3,17,18,19]. Among mechanical factors, non-anatomical femoral tunnel position is a highly debated factor associated with TW [20,21,22]. Ko et al. [21] reported that a more anterior and higher location of the femoral tunnel could be a risk factor for femoral TW. In contrast, Choi et al. [20] demonstrated that the femoral tunnel position is not a major factor associated with TW.

The graft of choice remains autograft for ACL reconstruction. Allografts have some advantages, including better cosmetic outcomes, lesser postoperative pain-related graft harvest, and faster recovery; however, they also have disadvantages, such as high cost and risk of TW [23,24]. Numerous studies have compared the postoperative outcomes between autografts and allografts [23,25,26]. However, only a few studies have investigated the risk factors for TW after ACL reconstruction using tibialis anterior allografts.

The present study aimed to identify potential factors for both femoral and tibial TW and to investigate the effect of TW on postoperative outcomes after ACL reconstruction with a tibialis anterior allograft. It was hypothesized that the tunnel position was associated with TW.

## 2. Materials and Methods

### 2.1. Study Design and Patients

**Figure 1:**
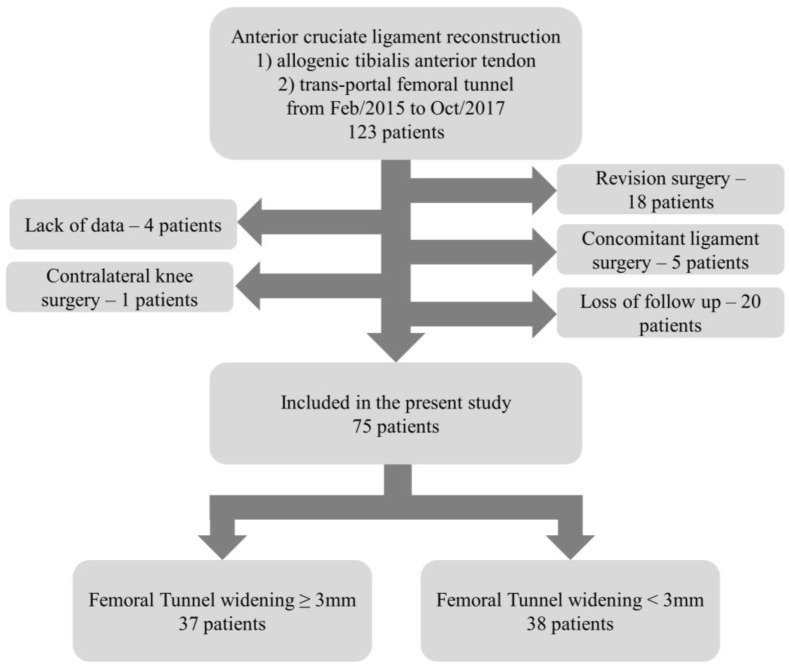
Flow chart of the enrolled patients.

This study was a retrospective comparative study. This study was approved by the ethics committee of our institution (SMC 2022-05-056), and written informed consent was obtained from all patients. The study enrolled patients who underwent arthroscopic ACL reconstruction at a single institution between February 2015 and October 2017 (Figure 1). The inclusion criteria were presented in Table 1. Overall, 123 patients were screened. Among them, a total 75 patients (75 knees) were enrolled after applying inclusion and exclusion criteria. The demographic, preoperative, and intraoperative findings are presented in Table 2. According to previous study results, the mean TW was 2.8–3.3 mm [11,27,28]. The patients were divided twice into two groups depending on whether the femoral or tibial TW was over or less than 3 mm on anteroposterior (AP) view radiographs.

### 2.2. Surgical Technique

**Figure 2:**
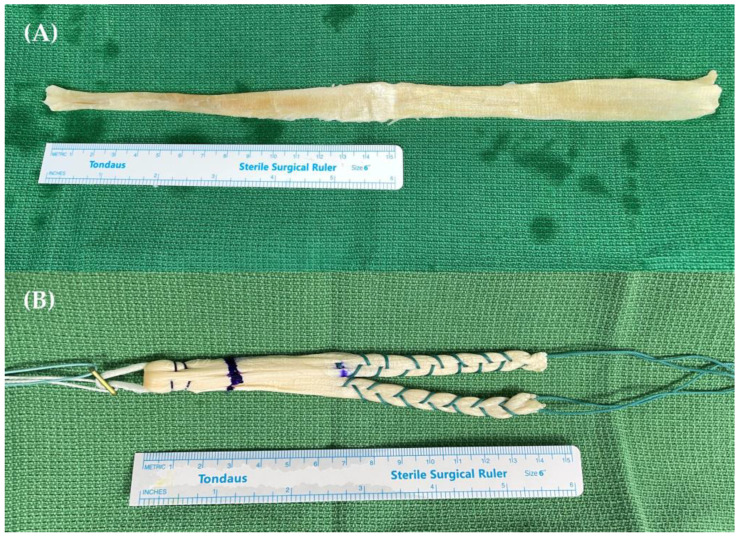
The allogenic tibialis anterior tendon (**A**) before and (**B**) after the preparation.

All surgeries were performed by a single senior surgeon, one of the authors (D.H.L). Arthroscopic portal formation and examinations were performed initially. Upon identifying a meniscal injury, an appropriate surgical procedure was performed depending on the tear characteristics before ACL reconstruction. After diagnosing an ACL injury, a femoral tunnel was created. The transanteromedial (AM) portal method was used to create the femoral tunnel at the anatomical position. A standard AM portal was used as the viewing portal, and the far AM portal was used as the working portal. A guide pin (2.4 mm) was inserted with knee flexion at 120°, and a 4.5 mm EndoButton drill (Smith & Nephew, Andover, MA, USA) was inserted to drill through the far cortex of the femur. After measuring the length, the femoral tunnel was created using a cannulated reamer. Subsequently, a tibial tunnel was created on the tibial footprint of the ACL. The allogeneic tibialis anterior tendon was prepared and grafted (Figure 2). An EndoButton (Smith & Nephew, Andover, MA, USA) was used for femoral side graft fixation. Hybrid fixation, which combines intra-tunnel aperture and extracortical suspensory fixation, was used for tibial side fixation [29].

Crutch-assisted walking was initiated 1 d after surgery. Full weight-bearing walking was permitted at six weeks. Range of motion (ROM) exercises were started from 0° to 90° 2 days after surgery, and full flexion was achieved by 6 weeks. Closed kinetic chain exercises were started two weeks postoperatively. Sports activity, including pivoting, jumping, or side-stepping, was allowed 9 months postoperatively.

### 2.3. Clinical and Radiographic Assessments

Clinical data were gathered in terms of ROM and patient-reported outcomes. The Lysholm score [30,31] and International Knee Documentation Committee (IKDC) score [32] were evaluated preoperatively and 2 years postoperatively. The pre- and postoperative clinical assessments were compared. Clinical outcomes were also compared between the groups divided into femoral or tibial TW ≥ 3 mm or <3 mm on AP radiographs.

**Figure 3:**
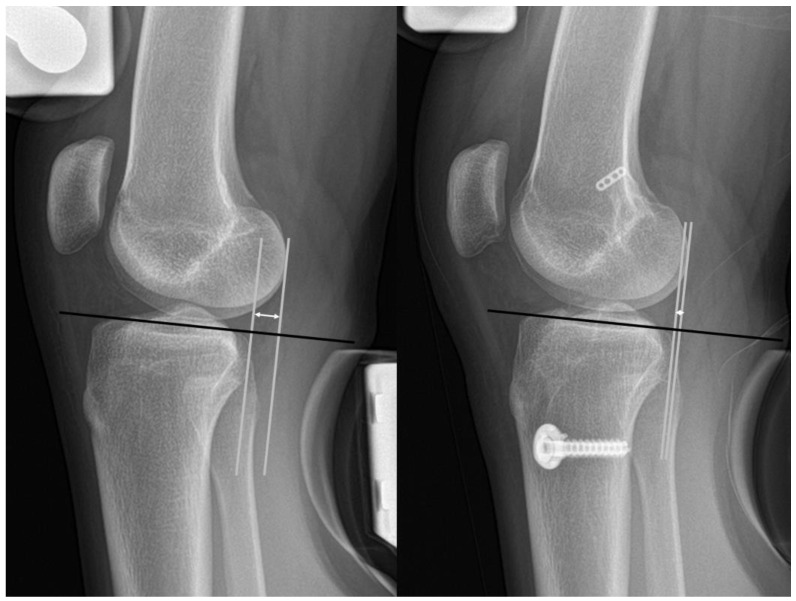
Measurement of pre- and postoperative anterior tibial translation on Telos stress radiographs. A reference line (black line) was drawn parallel to the tibial plateau joint line. Perpendicular lines (white lines) from the reference line were drawn tangentially to the most posterior contour of the femoral condyle and tibial plateau. The anterior tibial translation was defined as the distance between two lines (arrow line).

Postoperative plain radiographic outcomes were obtained from 2-year follow-up data. The preoperative hip-knee-ankle (HKA) angle was measured as the angle subtended by a line drawn from the center of the femoral head to the center of the knee, and a line drawn from the center of the knee to the center of the talus on whole-leg standing radiographs, with a positive and negative HKA angle indicating varus and valgus, respectively [33,34]. The tibial slope was measured as the angle between the mid-diaphysis line of the tibia and the line depicting the posterior inclination of the tibial plateau in the lateral view [35]. Anteroposterior knee joint stability was assessed using Telos Stress radiographs [36] (Figure 3). Preoperative and postoperative Telos Stress radiography (150 N on the tibia at 20–30° of knee flexion) was evaluated. A reference line was drawn parallel to the medial tibial plateau joint. The perpendicular lines from the reference line were drawn tangentially to the most posterior contour of the femoral condyle and the most posterior contour of the tibial plateau. The distance between the two lines was defined as anterior tibial translation. The side-to-side difference (STSD) was calculated to analyze native laxity, defined as the difference in anterior tibial translation between the knees. The STSD was measured preoperatively and at 2 years postoperatively, and the results were compared between each other.

**Figure 4:**
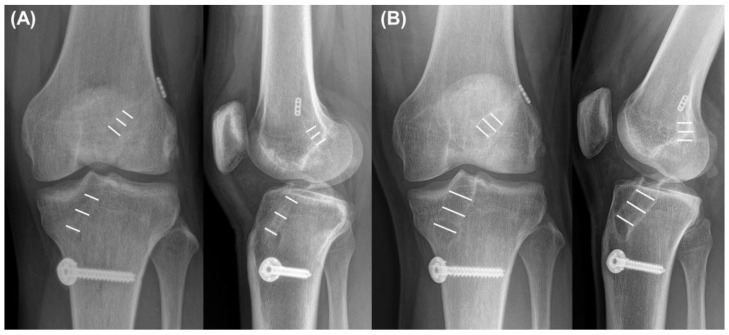
Measurement of (**A**) immediate and (**B**) 2-year postoperative tunnel diameters on the anteroposterior and lateral views. Tunnel widths were measured at three points on each tunnel—aperture, midpoint, and exit. Average values of each tunnel were used for analysis.

The tunnel widths of the femur and tibia were measured on both knee AP and lateral radiographs, as described in previous reports [28,37]. Immediately and 2 years postoperatively, radiographs were used to measure tunnel widths. The femoral and tibial tunnel widths on the AP (FT-AP and TT-AP, respectively) and lateral views (FT-Lat and TT-Lat, respectively) were measured (Figure 4). The average values of the three different measurement points were used for analysis [9]. Femoral and tibial TWs were defined as the measurement difference between immediate and postoperative results after 2 years on the AP view. The patients were divided into two groups according to femoral and tibial TWs > 3 mm.

**Figure 5:**
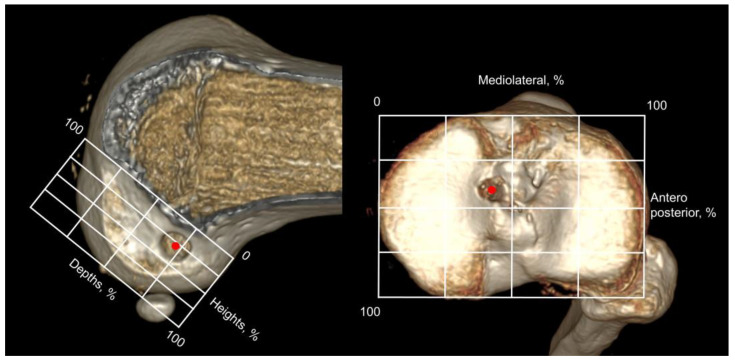
The center of the femoral and tibial tunnel (red dots) was calculated on 3-dimensional CT with the quadrant method. The height and depths of femoral tunnel position and anteroposterior and mediolateral tibial tunnel position were measured.

Computed tomography (CT) was performed 3 days postoperatively. Three-dimensional CT images were used to measure the femoral and tibial tunnel positions. The center of the femoral and tibial tunnel apertures was measured on the standardizeds grid system as described previously [20]. In terms of the femoral tunnel, the higher limit of the grid was located on the femoral notch roof, and the anterior, posterior, distal, and proximal sides of the grid were located on the articular cartilage margin. The height and depth of the femoral tunnel were measured. With respect to the tibial center, a rectangular grid was located at each end edge of the tibial plateau. The AP and mediolateral (ML) tibial tunnel positions were then calculated (Figure 5).

**Figure 6:**
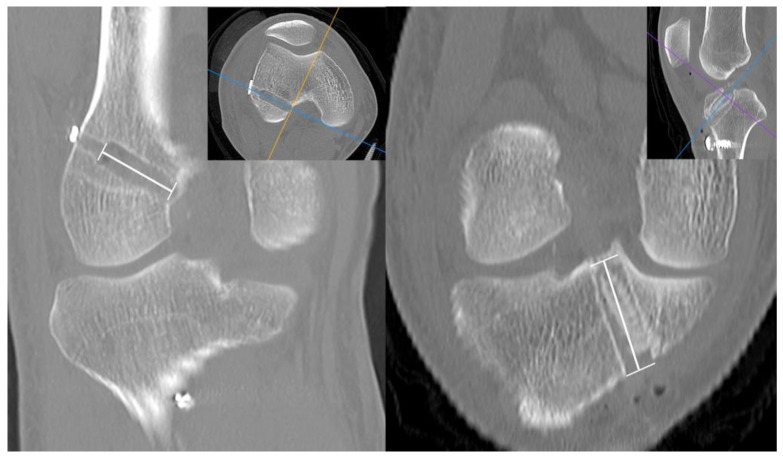
Measurement of the length of femoral and tibial tunnel on CT scan.

CT was also used to measure the femoral and tibial tunnel lengths. Images of the oblique plane which best visualized the long axis of the tunnels were analyzed using the Horos medical image viewer (version 3.3.5; Horos Project, New York, U.S.) [11] (Figure 6).

All radiographic parameters were measured twice by two orthopedic surgeons with at least 4-week intervals between each measurement using a picture archiving and communication system (Centricity PACS Viewer; GE Healthcare, Chicago, IL, USA). Intraclass correlation coefficients (ICC) were used to determine intraobserver and interobserver reliabilities.

### 2.4. Statistical Analysis

The Shapiro–Wilk test was used to evaluate the normality of the distribution. Paired *t*-tests for continuous variables and chi-square tests for categorical variables were used to compare the preoperative and postoperative outcomes. To compare the demographic data as well as preoperative, intraoperative, and postoperative outcomes between the two groups, Student’s *t*-test or the chi-square test was used. Stepwise multiple regression analysis was performed to identify which of the following factors were correlated with changes in the femoral and tibial TW on AP and lateral radiographs from the initial to 2-year follow-up. The independent factors were patient age, sex, body mass index, concomitant meniscal injury, HKA angle, tibial slope, femoral and tibial tunnel positions, and the length of both tunnels. All data were analyzed using IBM SPSS Statistics (version 27.0; IBM, Armonk, NY, USA), and statistical significance was set at *p* < 0.05. Our study allocated 37 and 38 patients to the femoral TW ≥ 3 mm and <3 mm groups, respectively. It would take 89% statistical power to detect a difference of at least 1 mm with a standard deviation of 1.5 mm in STSD between the femoral TW ≥ 3 mm and <3 mm groups (α = 0.05).

## 3. Results

All inter- and intraobserver ICCs showed good agreement with respect to the reliability of the radiographic measurements (>0.80).

Mean differences in femoral and tibial tunnel widths on AP and lateral radiographs between the immediate and 2-year postoperative periods were 3.0, 2.5, 2.9, and 2.9, respectively, in the following order: femoral tunnel on AP, femoral tunnel on the lateral, tibial tunnel on AP, and tibial tunnel on lateral. Detailed tunnel widths are summarized in Table 3.

When the patients were divided into two groups according to femoral TW ≥ 3 mm or <3 mm, 37 and 38 patients were classified into femoral TW ≥ 3 mm and <3 mm groups, respectively. Most preoperative, immediate, and postoperative outcomes were similar between the groups, except for the depths of the femoral tunnel position and postoperative STSD (Table 4).

The femoral tunnel position was significantly shallower in the femoral TW of ≥ 3 mm group. Postoperative STSD was significantly greater in the femoral TW ≥ 3 mm group. In terms of dividing groups as tibial TW ≥ 3 mm or <3 mm, 34 and 41 patients were classified as tibial TW ≥ 3 mm group and or < 3 mm group, respectively. No significant intergroup differences were observed in any outcome (Table 5).

In terms of the correlation between femoral TW on AP radiographs and potential predictive factors, the depths of femoral tunnel position (shallow position of the femoral tunnel) were significantly correlated with femoral TW on AP radiographs (adjusted *R*^2^ = 0.134, Table 6). The correlation between femoral TW on lateral radiograph and potential factors showed similar results; a shallower femoral tunnel is the most critical predictive factor for femoral TW on the lateral radiograph (adjusted *R*^2^ = 0.176, Table 7). Regarding the correlation between tibial TW on AP and lateral radiographs and potential predictive factors, all factors were not significantly related to tibial TW.

**Figure 7:**
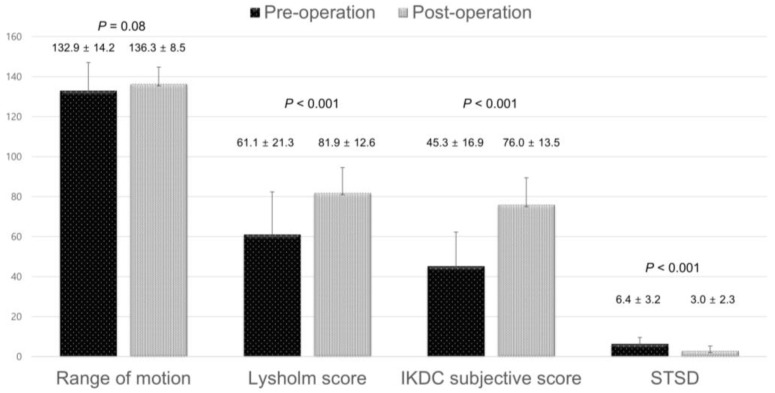
Comparison of clinical outcomes and side-to-side difference on stress radiographs between pre- and post-operation.

The Lysholm and IKDC subjective scores significantly improved postoperatively. STSD on Telos stress radiographs was significantly less postoperatively compared to pre-operation (Figure 7).

## 4. Discussion

The principal finding of the present study was that the shallow position of the femoral tunnel was correlated with femoral TW after ACL reconstruction using a tibialis anterior allograft. Moreover, the femoral TW ≥ 3 mm group showed inferior postoperative anterior stability than the femoral TW < 3 mm group.

TW after ACL reconstruction was widely reported in the early 1990s [14,38]. Despite its wider recognition and numerous previous studies, there is little information available on the origin and reason for bone TW. Clatworthy et al. [2] suggested a multifactorial etiology of tunnel enlargement. It is generally agreed that TW after ACL reconstruction occurs because of a complex interplay between mechanical and biological factors. In terms of mechanical factors, the key terms were ‘motion of grafts’ and ‘strain of grafts’ in the tunnels. For example, cortical suspensory device fixation provided greater graft motion at the aperture area, such that TW could be greater than aperture fixation [7,39]. Young age and male sex were reportedly risk factors for TW in previous studies, suggesting that greater activity might induce greater motion of grafts in tunnels [9,12]. A greater tibial slope was reported as a risk factor for tibial TW in a previous study [40]. They guessed that an increased tibial slope induced anterior translation of the tibia during weight-bearing activities, potentially placing more strain on the graft, resulting in increased TW. In a brief review of previous studies investigating biological factors, Zijl et al. [28] compared tunnel enlargement between bone-patellar tendon-bone (BPTB) autografts and allografts. The average tunnel enlargement was found to be 2.2 mm ± 2.5 mm for autografts and 2.8 mm ± 2.1 mm for allografts without a statistically significant difference. Fahey et al. [14] also compared BPTB autografts and allografts; the average TW was 0.26 mm for autografts and 1.2 mm for allografts with significance (*p* > 0.0002). Zhang et al. [41] compared the ratio of tibial tunnel enlargement (compared to initial tunnel width) between hamstring autograft and soft tissue allograft, including tibialis and hamstring. The ratio of tibial tunnel enlargement was 26.7% ± 4.0% for autografts and 29.7% ± 5.3% for allografts, which was statistically significant (*p* = 0.009). Amano et al. [42] compared the ratio of femoral tunnel enlargement between hamstring autografts and BPTB autografts. The ratio of femoral tunnel enlargement was 41.9% ± 22.2% for the hamstring tendon and 16.0% ± 12.4% for the allografts, with a statistically significant difference (*p* < 0.05). Kim et al. [43] compared the ratio of femoral tunnel enlargement between Achilles tendon allografts and tibialis anterior allografts. The ratio of femoral tunnel enlargement was 38.4% ± 35.8% for the tibialis anterior allografts and 16.1% ± 17.6% for the tibialis anterior allografts (*p* = 0.017). The results so far imply that allografts have a slight disadvantage for TW compared to autografts, and TW mainly occurred in bone-soft tissue contact surfaces rather than bone-bone contact surfaces in both allo- and autografts. Therefore, it is important to investigate the predictive factors of TW after ACL reconstruction using hamstring or tibialis allografts. Only a few studies have investigated predictive factors for TW after surgery with hamstring or tibialis allografts. Moon et al. [11] studied 91 patients who underwent ACL reconstruction using a tibialis anterior allograft with suspensory femoral fixation. They conducted multiple regression analyses between the potential independent factors and femoral TW. They concluded that a short graft insertion length (β = −1.724, *p* < 0.001) and shallow femoral tunnel position (β = 0.407, *p* = 0.008) were associated with femoral TW. The relationship between the femoral TW and femoral tunnel position was consistent with our results. However, the femoral tunnel length, similar to the graft insertion length in the study by Moon et al., was not correlated with the femoral TW in our study. Our study and the study by Moon et al. were relatively small-volume investigations; therefore, a large-volume study is needed to identify risk factors for TW after ACL reconstruction using allografts more precisely.

The importance of ACL reconstruction in anatomical tunnel positions is widely known. Parkinson et al. [44] reported that the femoral center of the ACL footprint was defined as 29.3% ± 3.5% in the anteroposterior plane (shallow–deep) and 34.7% ± 4.5% in the proximal-distal plane (high–low). Previous biomechanical cadaveric studies suggested that knees with grafts placed anterior and proximal to the anatomic femoral footprint experienced more anterior tibial translation and less rotational stability [45,46]. Similar to the results of biomechanical studies, previous studies demonstrated that anatomical femoral tunnel position was correlated with better anterior and rotational stability than non-anatomical ACL reconstruction [10,22,47]. Moreover, Byrne et al. [48] reported that non-anatomical femoral tunnel position was an independent risk factor for revision ACL reconstruction. It remains unclear whether the non-anatomical femoral tunnel position, especially in shallow and high positions, is correlated with femoral TW [11,20,21]. In our study, the shallow femoral tunnel position was correlated with femoral TW. Previous studies demonstrated that ACL grafts were positioned eccentrically to shallow and high positions and filled 52.0–55.3% of the femoral tunnel when femoral fixation was performed using a cortical suspensory device [7,49]. Despite these inconsistent findings, we believe the femoral tunnel position would be an independent risk factor for femoral TW after ACL reconstruction using the cortical suspensory device aspect of graft motion and strain.

Whether TW is associated with knee laxity after ACL reconstruction remains controversial [6,8]. The femoral TW ≥ 3 mm group showed inferior knee anterior stability compared to the <3 mm group in the present study. The femoral tunnel position was also significantly shallower in the femoral TW of ≥ 3 mm group. Therefore, it is challenging to determine TW as an independent relative factor for postoperative knee instability by analyzing our results. We believe that TW, non-anatomic tunnel position, and knee laxity may be related to each other. A large-volume study is needed to further identify the relationship between TW and postoperative knee laxity.

Our study has several limitations. First, the strength of this study is that all surgeries were performed by a single surgeon using the same graft, fixation method, and tunnel formation technique. However, our results cannot be applied to other graft selections, tunnel formation methods, or fixation methods. Second, the patients were divided into TW ≥ 3 mm and TW < 3 mm groups according to the change in tunnel width from the initial to 2-year postoperatively. Furthermore, the number of patients varied if the follow-up period was different. Therefore, a causal relationship may not be strong. Third, the tunnel width was measured using plain radiographs; therefore, the measurements could be inaccurate compared with those obtained from CT or magnetic resonance imaging. Fourth, this study had a retrospective design; therefore, selection or information biases may exist.

## 5. Conclusions

The shallow position of the femoral tunnel was correlated with femoral TW after ACL reconstruction using a tibialis anterior allograft. A femoral TW ≥ 3 mm showed inferior postoperative knee anterior stability. However, postoperative clinical outcomes were similar between both groups. Postoperative clinical outcomes were significantly improved compared to those of preoperation.

## Figures and Tables

**Table 1:** Inclusion and exclusion criteria of the study.

**Inclusion Criteria**
(1) total ACL rupture diagnosed during magnetic resonance imaging or arthroscopic examination
(2) ACL reconstruction using a tibialis anterior allograft
(3) follow-up for more than 24 months
**Exclusion criteria**
(1) revision ACL reconstruction
(2) concomitant ligament surgery
(3) history of contralateral knee surgery
(4) lack of clinical or radiographic data

**Table 2:** Demographic, preoperative, and intraoperative data for enrolled patients.

Number of Patients	75
Age, year	31.5 ± 12.0 (18–60)
Sex, M:F	61:14:00
Body mass index, kg/m^2^	24.9 ± 3.7 (18.5–34.7)
Side of injury, Right:Left	42:33:00
Combined meniscus injury, *n* (%)	
Medial	33 (44%)
Lateral	10 (13.3%)
Both	7 (9.3%)
HKA angle, degree	0.5 ± 2.56 (−5.8–9.2)
Tibial slope, degree	10.8 ± 3.3 (3.1–17.8)
Femoral tunnel length, mm	29.5 ± 4.8 (20–40)
Tibial tunnel length, mm	35.0 ± 5.2 (24–48)
Femoral tunnel position, %	
Depth	29.6 ± 7.0 (17–53)
Height	33.3 ± 8.7 (5–48)
Tibia tunnel position, %	
Anteroposterior	44.0 ± 2.6 (36–51)
Mediolateral	42.8 ± 6.6 (29–54)

Data are presented as mean ± standard deviation (range); HKA, hip-knee-ankle.

**Table 3:** Tunnel widths on anteroposterior and lateral knee radiographs at immediate and 2-year post-operation.

	Immediately after Operation	2-Year after Operation	*p* Value
Femoral tunnel width on AP radiograph, mm	10.1 ± 1.0	13.1 ± 1.8	<0.001
Femoral tunnel width on lateral radiograph, mm	9.9 ± 1.2	12.4 ± 1.8	<0.001
Tibial tunnel width on AP radiograph, mm	10.3 ± 0.9	13.2 ± 1.4	<0.001
Tibial tunnel width on lateral radiograph, mm	10.7 ± 1.1	13.6 ± 1.5	<0.001

**Table 4:** Comparison of preoperative, immediate and postoperative outcomes between femoral tunnel widening ≥3 mm group and <3 mm group.

	Femoral TW ≥ 3 mm	Femoral TW < 3 mm	*p*-Value
Number of patients	37	38	
Age, year	33.1 ± 13.5	29.9 ± 10.3	0.257
Sex, male:female	30:07:00	31:07:00	0.956
Body mass index, kg/m^2^	24.9 ± 3.9	24.9 ± 3.5	0.975
Direction, right:left	21:16	21:17	0.896
Combined meniscal injury, *n*			
Medial meniscus	17	16	0.902
Lateral meniscus	5	5	0.831
Both medial and lateral	3	4	0.978
Hip-knee-ankle angle, °	0.8 ± 2.6	0.1 ± 2.5	0.218
Tibial slope, °	11.2 ± 3.6	10.4 ± 3.0	0.272
Femoral tunnel length, mm	29.5 ± 5.0	29.5 ± 4.7	0.98
Tibial tunnel length, mm	35.8 ± 5.5	33.9 ± 4.7	0.105
Femoral tunnel position—Depth, %	31.7 ± 7.3	27.6 ± 6.3	0.014
Femoral tunnel position—Height, %	32.8 ± 8.8	33.6 ± 8.8	0.638
Tibia tunnel position—AP, %	43.6 ± 6.9	42.0 ± 6.3	0.288
Tibia tunnel position—ML, %	44.1 ± 2.6	44.0 ± 2.6	0.751
Preoperative ROM, °	130.7 ± 15.5	128.2 ± 15.2	0.546
Preoperative Lysholm score	62.0 ± 24.0	55.7 ± 17.8	0.33
Preoperative IKDC subjective score	44.9 ± 20.1	42.6 ± 14.3	0.671
Preoperative STSD, mm	6.4 ± 2.7	6.5 ± 3.6	0.949
Postoperative 2-year ROM, °	138.4 ± 9.3	136.5 ± 8.5	0.44
Postoperative 2-year Lysholm score	84.1 ± 12.1	87.0 ± 11.8	0.318
Postoperative 2-year IKDC subjective score	78.4 ± 12.3	76.6 ± 14.6	0.583
Postoperative 2-year STSD, mm	3.7 ± 2.0	2.4 ± 2.3	0.013

AP, anteroposterior; ML, mediolateral; ROM, range of motion; STSD, side-to-side difference.

**Table 5:** Comparison of preoperative, immediate, and postoperative outcomes between tibial tunnel widening ≥3 mm group and < 3 mm group.

	Tibial TW ≥ 3 mm	Tibial TW < 3 mm	*p*-Value
Number of patients	34	41	
Age, year	21.2 ± 12.0	30.9 ± 12.1	0.651
Sex, male:female	26:08:00	35:06:00	0.381
Body mass index, kg/m^2^	24.8 ± 4.2	25.0 ± 3.2	0.849
Direction, right:left	19:15	23:18	0.985
Combined meniscal injury, *n*			
Medial meniscus	15	18	0.598
Lateral meniscus	5	5	0.786
Both medial and lateral	2	5	0.813
Hip-knee-ankle angle, °	0.7 ± 2.3	0.3 ± 2.7	0.494
Tibial slope, °	11.4 ± 3.8	10.3 ± 2.9	0.153
Femoral tunnel length, mm	29.5 ± 3.3	29.5 ± 5.8	0.96
Tibial tunnel length, mm	34.8 ± 5.5	34.9 ± 4.9	0.971
Femoral tunnel position—Depth, %	29.0 ± 6.9	30.1 ± 7.3	0.544
Femoral tunnel position—Height, %	34.1 ± 8.7	32.6 ± 8.7	0.465
Tibia tunnel position—AP, %	44.0 ± 6.6	41.8 ± 6.5	0.15
Tibia tunnel position—ML, %	44.1 ± 2.6	44.0 ± 2.7	0.88
Preoperative ROM, °	131.7 ± 12.6	127.5 ± 15.5	0.324
Preoperative Lysholm score	62.5 ± 24.4	55.2 ± 17.0	0.258
Preoperative IKDC subjective	44.0 ± 19.5	43.5 ± 15.3	0.932
Preoperative STSD, mm	6.7 ± 4.0	6.2 ± 2.3	0.426
Postoperative 2-year ROM, °	136.6 ± 8.9	138.3 ± 8.9	0.489
Postoperative 2-year Lysholm score	83.5 ± 13.9	87.4 ± 9.9	0.184
Postoperative 2-year IKDC subjective score	76.3 ± 13.5	78.4 ± 13.5	0.535
Postoperative 2-year STSD, mm	3.6 ± 2.1	2.6 ± 2.3	0.055

AP, anteroposterior; ML, mediolateral; ROM, range of motion; STSD, side-to-side difference.

**Table 6:** Multiple regression analysis of potential predictive factors correlated with the femoral tunnel widening observed on the anteroposterior radiograph.

Dependent Variable	Independent Variables	Non-Standardized Coefficients		Standardized Coefficients	*p*-Value
		B	SE	B	
Femoral tunnel widening on AP radiograph (Initial→2 years)	Age	0.014	0.018		0.238
	Sex	−0.146	0.649		0.567
	Body mass index	0.001	0.061		0.696
	Medial meniscus injury	−0.085	0.434		0.751
	Lateral meniscus injury	−0.225	0.597		0.635
	Hip-knee-ankle angle	0.081	0.085		0.173
	Tibial slope	−0.012	0.073		0.724
	Femoral tunnel length	−0.007	0.051		0.981
	Tibial tunnel length	0.007	0.059		0.536
	Femoral tunnel position—Depth	8.813	3.798	0.387	0.003
	Femoral tunnel position—Height	1.355	2.597		0.485
	Tibia tunnel position—AP	0.54	9.384		0.613
	Tibia tunnel position—ML	3.527	4.272		0.39

AP, anteroposterior; ML, mediolateral.

**Table 7:** Multiple regression analysis of potential predictive factors correlated with the femoral tunnel widening observed on the lateral radiograph.

Dependent Variable	Independent Variables	Non-Standardized Coefficients		Standardized Coefficients	*p*-Value
		B	SE	B	
Femoral tunnel widening on the lateral radiograph (Initial→2 years)	Age	−0.009	0.015		0.524
	Sex	−0.456	0.538		0.401
	Body mass index	0.002	0.051		0.972
	Medial meniscus injury	−0.097	0.36		0.789
	Lateral meniscus injury	−0.204	0.495		0.681
	Hip-knee-ankle angle	0.129	0.071		0.077
	Tibial slope	−0.044	0.061		0.478
	Femoral tunnel length	−0.054	0.042		0.21
	Tibial tunnel length	−0.039	0.049		0.435
	Femoral tunnel position—Depth	8.462	2.348	0.437	0.001
	Femoral tunnel position—Height	0.221	2.153		0.919
	Tibia tunnel position—AP	−5.223	7.779		0.506
	Tibia tunnel position—ML	2.672	3.541		0.455

AP, anteroposterior; ML, mediolateral.

## Data Availability

Not applicable.
